# Environmental Lead Exposure Predicts Lower Bone Mineral Density in a Large Taiwanese Population-Based Study

**DOI:** 10.7150/ijms.117042

**Published:** 2025-09-08

**Authors:** Po-Yin Shen, Fu-Wen Liang, Cheng-Chang Lu, Shu-Pin Huang, Szu-Chia Chen, Jiun-Hung Geng

**Affiliations:** 1 Department of Orthopaedics, Kaohsiung Municipal Siaogang Hospital, Kaohsiung 812, Taiwan.; 2 Department of Orthopaedics, Kaohsiung Medical University Hospital, Kaohsiung Medical University, Kaohsiung 807, Taiwan.; 3 School of Medicine, College of Medicine, Kaohsiung Medical University, Kaohsiung 807, Taiwan.; 4 Department of Public Health, College of Health Sciences, Kaohsiung Medical University, Kaohsiung 807, Taiwan.; 5 Department of Medical Research, Kaohsiung Medical University Hospital, Kaohsiung Medical University, Kaohsiung 807, Taiwan.; 6 Center for Big Data Research, Kaohsiung Medical University, Kaohsiung 807, Taiwan.; 7 Department of Urology, Kaohsiung Medical University Hospital, Kaohsiung Medical University, Kaohsiung 807, Taiwan.; 8 Department of Urology, School of Medicine, College of Medicine, Kaohsiung Medical University 807, Kaohsiung, Taiwan.; 9 Graduate Institute of Clinical Medicine, College of Medicine, Kaohsiung Medical University, Kaohsiung 807, Taiwan.; 10 Research Center for Environmental Medicine, Kaohsiung Medical University, Kaohsiung 807, Taiwan.; 11 Ph.D. Program in Environmental and Occupational Medicine, College of Medicine, Kaohsiung Medical University, Kaohsiung 807, Taiwan.; 12 Institute of Medical Science and Technology, College of Medicine, National Sun Yat-Sen University, Kaohsiung 804, Taiwan.; 13 Department of Internal Medicine, Kaohsiung Municipal Siaogang Hospital, Kaohsiung Medical University, Kaohsiung 812, Taiwan.; 14 Division of Nephrology, Department of Internal Medicine, Kaohsiung Medical University Hospital, Kaohsiung Medical University, Kaohsiung 807, Taiwan.; 15 Faculty of Medicine, College of Medicine, Kaohsiung Medical University, Kaohsiung 807, Taiwan.; 16 Department of Urology, Kaohsiung Municipal Siaogang Hospital, Kaohsiung 812, Taiwan.

**Keywords:** epidemiologic study, osteoporosis, lead, Taiwan biobank, bone mineral density

## Abstract

**Background:** Chronic exposure to environmental lead (Pb) may impair bone metabolism and increase the risk of osteoporosis; however, large-scale epidemiological data remain limited. This study aimed to examine the association between long-term environmental Pb exposure and bone mineral density (BMD) in a nationally representative Taiwanese cohort.

**Methods:** A total of 32,239 participants aged 30-70 years were included after excluding those with missing lead exposure or BMD data. BMD was assessed via calcaneal quantitative ultrasound, and T-scores were calculated. Environmental Pb exposure was estimated using a validated Geo-AI model. Univariable and multivariable linear regression models were used to evaluate associations between Pb exposure and T-scores, adjusting for demographic, lifestyle, comorbidity, and laboratory factors.

**Results:** Among all participants, 7.2% had T-scores < -2.5. These individuals were older, had lower educational attainment, and demonstrated less favorable metabolic and clinical profiles. Multivariable stepwise regression analysis revealed a significant inverse association between lead exposure and baseline T-scores: each 0.001 µg/m³ increase in airborne Pb was independently associated with a -0.013 decrease in baseline T-score (95% CI: -0.017 to -0.009, p < 0.0001). More importantly, in longitudinal analyses, each 0.001 µg/m³ increase in Pb concentration was independently associated with a -0.006 decrease in within-individual T-score change over time (95% CI: -0.010 to -0.003, p = 0.0002). Other significant factors influencing T-scores and T-score changes included age, sex, body mass index, education, exercise, and albumin levels.

**Conclusions:** Chronic environmental Pb exposure is significantly associated with reduced bone mineral density in the general population. These findings underscore the need for environmental risk assessments in osteoporosis screening and public health prevention strategies, particularly in vulnerable populations.

## Introduction

Lead (Pb), a pervasive toxic heavy metal, poses a significant global health concern due to its widespread presence in the environment and numerous human-made products, including petrol additives, lead-based paints, and even some cookware and construction materials. Human exposure occurs through various pathways, leading to the accumulation of lead in the body, with a striking 94% stored in bones, where it can disrupt normal bone mineral deposition, development, and maturation 1, 2. The World Health Organization has recognized the severe toxicological impact of lead, linking it to hundreds of thousands of deaths annually 3.

Osteoporosis, a systemic skeletal disease affecting approximately 200 million people globally and causing millions of fractures each year, represents a major public health burden characterized by reduced bone mass and increased fracture risk 4. While traditional risk factors like age, gender, and genetics are well-established, emerging evidence suggests that environmental toxins—particularly Pb—may also contribute to the disease's progression 5, 6. Lead's ability to substitute calcium in bone and interfere with bone cell function provides a biologically plausible link to impaired bone health 7, 8.

Although in vitro and animal studies have indicated a connection between lead exposure and reduced bone density and strength 2, 9, the precise impact of lead exposure on the rate of bone loss in diverse human populations remains less understood. Population-based studies with longitudinal data are crucial to clarify this relationship. This study aims to address this critical knowledge gap by examining the effects of lead exposure on both bone mineral density (BMD) and, importantly, the rate of bone loss over time. Utilizing comprehensive longitudinal data from over 19,000 participants in the Taiwan Biobank (TWB), which includes detailed demographic, lifestyle, and environmental information, this research offers a unique and comprehensive evaluation of lead's long-term impact on bone health. The findings from this study have the potential to inform public health strategies aimed at mitigating the detrimental effects of lead exposure on skeletal health.

## Materials and Methods

### Study design and population

**Figure 1:**
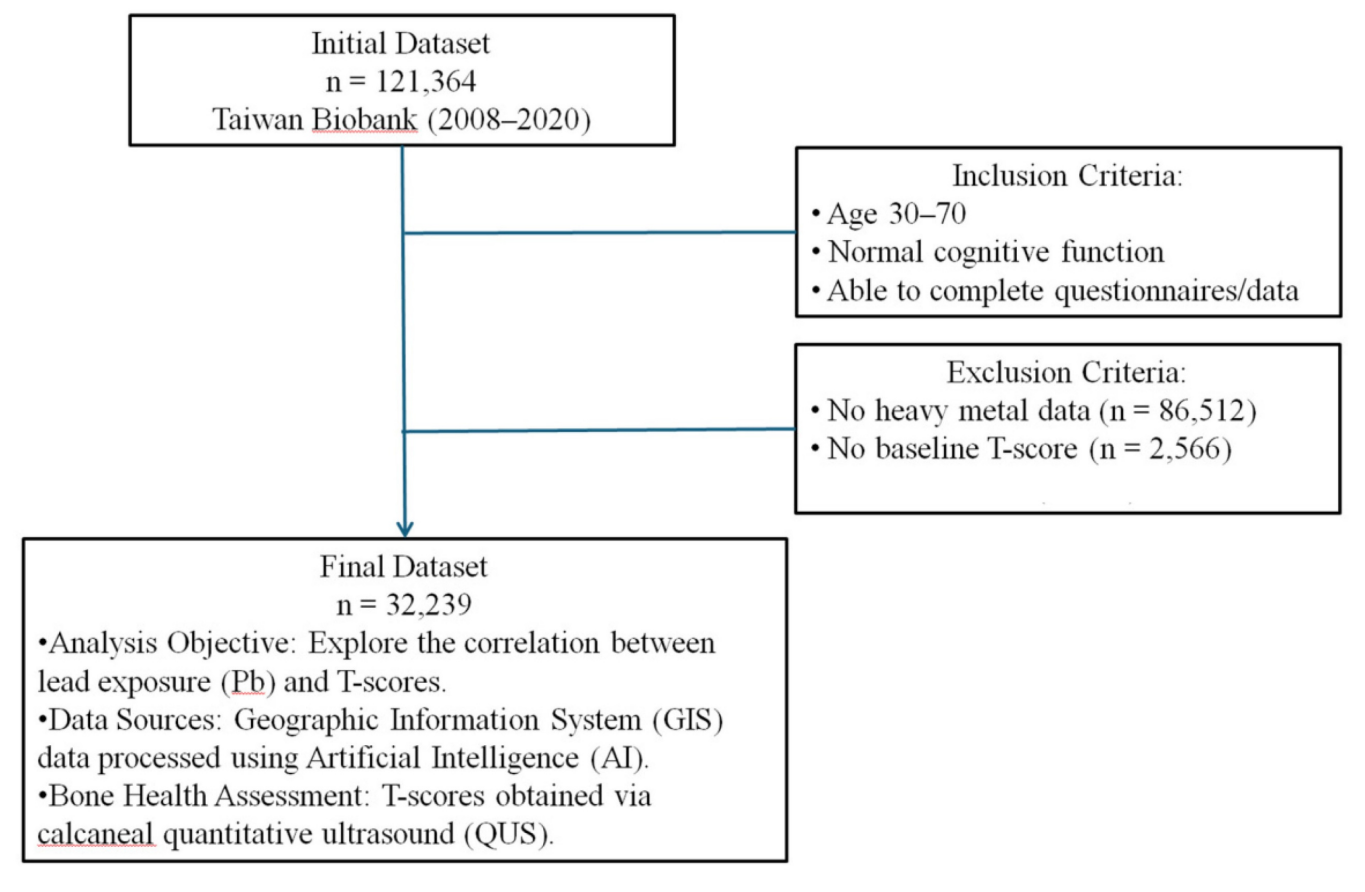
Study framework and participant selection process. Ascertainment of T-score and definition of osteoporosis.

This cohort study utilized data collected by the TWB from 2008 to 2020 10-12. Ethical approval was obtained from the Ethics and Governance Council of the TWB and the Institutional Review Board of Kaohsiung Medical University (approval number KMUHIRB-E(I)-20210058)], covering the study period from April 8, 2021, to March 31, 2026. Informed consent was obtained from all participants. The TWB database includes 121,364 participants. Inclusion criteria were adults aged 30-70 years with normal cognitive function, able to understand and complete the questionnaire and data collection process. Participants with a baseline diagnosis of osteoporosis, missing BMD data, Pb exposure data, or essential demographic information were excluded, resulting in 32,239 participants for the final analysis (**Figure 1**).

Eligible participants provided comprehensive information on residential addresses, age, gender, height, weight, smoking habits, alcohol consumption, educational status, and medical history, including conditions such as hypertension, diabetes mellitus, dyslipidemia, and cataract. Participants were followed for an average of 43 months (range: 2-4 years between BMD measurements), and calcaneal T-scores were measured repeatedly in the same individuals at different time points. The “T-score changes” used in our longitudinal analyses represent within-individual differences between follow-up and baseline measurements, enabling us to directly assess changes in T-score over time.

### BMD measurements

BMD was assessed using calcaneal quantitative ultrasound (QUS) with the Achilles InSight device (GE, Madison Heights, USA). This method was selected for its portability, non-invasive nature, low cost, and absence of radiation exposure, making it suitable for large-scale population studies. The calcaneus, chosen for its high trabecular content and metabolic turnover rate, offers a bone structure similar to that of the spine, allowing for reliable osteoporosis risk prediction. Using QUS, T-scores (g/cm²) were derived from the calcaneal BMD by comparing each participant's BMD to the mean BMD of young adults, with osteoporosis diagnosed at T-scores ≤ -2.5 SD below the young adult reference value 13.

### Environmental lead assessments

Environmental Pb exposure was assessed using a cutting-edge geospatial artificial intelligence (Geo-AI) system developed by Dr. Wu and colleagues 14-16. This system integrates high-resolution spatial data with machine learning algorithms to model ambient heavy metal concentrations across Taiwan. Specifically, the Geo-AI framework combines geographic information system (GIS) data, such as land use patterns, proximity to industrial zones, traffic density, thermal power plant locations, and road networks, with satellite-derived imagery and meteorological variables.

Lead concentration estimates were derived from Taiwan Environmental Protection Administration (EPA) air quality data collected at 74 fixed-site monitoring stations nationwide from 1994 to 2020. These stations employed standardized instrumentation to continuously measure airborne lead levels, and historical data were used to train ensemble learning models including CatBoost, XGBoost, Random Forest Regression (RFR), Gradient Boosting Machines (GBM), and LightGBM.

The Geo-AI model generated temporally and spatially resolved lead concentration maps with a 50 m × 50 m resolution, capable of capturing seasonal and diurnal variations. Each participant's long-term environmental lead exposure was determined by linking their residential address to the modeled Pb concentration in their corresponding geographic grid cell. For this study, we used the 1-year average Pb concentration prior to the baseline assessment to represent individual-level exposure.

### Covariates

Demographic (age, sex, education), lifestyle (smoking, alcohol consumption, physical activity), anthropometric (body mass index [BMI], waist circumference), and clinical variables (hypertension, diabetes, dyslipidemia, cataract) were collected via standardized questionnaires and medical examinations. Laboratory data included hemoglobin, albumin, total cholesterol, uric acid, systolic and diastolic blood pressure.

### Statistical analysis

Descriptive statistics were used to compare participant characteristics by osteoporosis status (T-score ≤ -2.5 vs. > -2.5). Continuous variables were expressed as means ± standard deviations and compared using independent t-tests; categorical variables were compared using Chi-square tests. Multivariable linear regression and stepwise regression models were constructed to assess the association between environmental lead exposure and T-scores, adjusting for potential confounders. For longitudinal analyses, additional regression models evaluated within-individual changes in T-score over time using repeated follow-up measurements from the same participants. To assess the validity of the stepwise multivariable linear regression model, we performed comprehensive regression diagnostics. Homoscedasticity was evaluated using the Breusch-Pagan test. In the presence of heteroscedasticity, heteroscedasticity-consistent (HC3) standard errors were calculated. Multicollinearity was assessed using variance inflation factors (VIF), with values < 5 considered acceptable. Influential observations were identified using Cook's distance. Residual patterns were examined using residuals-vs-fitted plots, and normality of residuals was assessed using Q-Q plots. To explore potential effect modification by age and sex, stratified analyses were conducted. Age was dichotomized at 50 years (>50 vs. ≤50 years), and sex was categorized as female or male. Within each stratum, unadjusted and multivariable-adjusted linear regression models were fitted using the same covariates as in the primary analysis. Regression coefficients, 95% confidence intervals (CI), and p-values were reported. A two-sided p-value < 0.05 was considered statistically significant. All analyses were performed using SPSS version 19.0 (SPSS Inc., Armonk, NY) and R version 4.2.2 (R Foundation for Statistical Computing, Vienna, Austria).

## Results

### Participant characteristics

A total of 32,239 participants were included in the analysis, with a mean age of 49.7 ± 11.3 years. Based on bone mineral density, participants were stratified into two groups: T-score ≥ -2.5 (n = 29,924) and T-score < -2.5 (n = 2,315). The overall mean T-score was -0.3 ± 1.6; group-specific means were -0.1 ± 1.5 for those with T-scores ≥ -2.5 and -3.1 ± 0.5 for those with T-scores < -2.5 (p < 0.0001).

Participants with T-scores < -2.5 were older (58.1 ± 9.2 vs. 49.1 ± 11.2 years, p < 0.0001) and less likely to be female (60.1% vs. 64.4%, p < 0.0001). Educational attainment significantly differed between groups, with individuals in the low bone density group less likely to have completed university or higher education compared to those with normal bone density (50.4% vs. 67.8%, p < 0.0001). Regular exercise was less common in this group (37.8% vs. 47.4%, p < 0.0001), while smoking prevalence was slightly higher (29.8% vs. 27.4%, p = 0.016).

Comorbidities including diabetes, hypertension, dyslipidemia, and cataract were significantly more prevalent in participants with T-scores < -2.5 (all p < 0.0001). Laboratory measures such as systolic and diastolic blood pressure, total cholesterol, and albumin also differed significantly between groups. Full demographic and clinical characteristics stratified by bone density status are summarized in **Table 1**.

### Association between environmental lead exposure and T-score

In the univariable linear regression analysis (Table 2), each increase of 0.001 µg/m³ in airborne Pb exposure was significantly associated with a lower baseline T-score (β = -0.008, 95% CI: -0.013 to -0.004, p = 0.0003). Other factors negatively associated with T-score included older age, absence of regular exercise, higher waist circumference, lower BMI, current smoking, current alcohol consumption, and several comorbidities such as diabetes, hypertension, dyslipidemia, and cataract. Laboratory parameters including elevated systolic and diastolic blood pressure, total cholesterol, and higher levels of hemoglobin and uric acid were also linked to lower T-scores. In contrast, female sex, higher education, and serum albumin were positively associated with T-score.

In the multivariable model (Table 3), after adjusting for relevant covariates, airborne lead exposure remained significantly and inversely associated with baseline T-score. Specifically, multivariable stepwise regression analysis showed that each 0.001 µg/m³ increase in airborne Pb was independently associated with a -0.013 decrease in baseline T-score (β = -0.013, 95% CI: -0.017 to -0.009, p < 0.0001), and this association persisted across both the full and stepwise models. Additional independent predictors of lower T-score included older age, greater waist circumference, and lower BMI, while female sex, university education, and regular exercise were associated with higher T-scores. Diabetes showed a weak positive association, whereas cataract and higher levels of systolic blood pressure, total cholesterol, and serum albumin were independently linked to lower T-scores.

### Parameters associated with longitudinal changes in T-score

Participants were followed for an average of 43 months, with repeat bone density measurements. The change in T-score (ΔT-score) was modeled to evaluate the effects of baseline covariates on bone loss over time, representing within-individual longitudinal changes. As shown in Table 4, univariable regression identified that each 0.001 µg/m³ increase in Pb exposure predicted a ΔT-score decrease of -0.003 (95% CI: -0.006 to 0.000, p = 0.0847), which approached statistical significance. In multivariable stepwise regression, the association became both stronger and statistically significant: each 0.001 µg/m³ increase in air lead was independently associated with a further decline in T-score (β = -0.006, 95% CI: -0.010 to -0.003, p = 0.0002), after adjusting for other longitudinal confounders.

We conducted regression diagnostics to validate the model. The BP test indicated heteroscedasticity (BP statistic = 140.26, p < 0.001), so HC3 standard errors were applied, and the association between lead exposure and T-score change remained highly significant (p <0.001; Supplementary Table S1). All VIF values were < 2, indicating no multicollinearity (Supplementary Table S2). Cook's distance (< 0.02) showed no influential points (Supplementary Figure S1), and residual plots supported linearity and reasonable homoscedasticity (Supplementary Figure S2). Q-Q plots showed minor tail deviations but were generally consistent with normality (Supplementary Figure S3). Other factors independently contributing to greater T-score decline included longer follow-up time, older age, and increased waist circumference, while higher BMI and higher education level were associated with lower rates of T-score decline. Notably, traditional risk factors such as gender, regular exercise, and several laboratory parameters were not retained in the final stepwise model of longitudinal change.

### Parameters associated with longitudinal changes in T-score in subgroup analyses by age and sex

Stratified models showed that higher lead exposure was associated with greater declines in T-score across all strata, except for males in the unadjusted model. The effect was stronger in participants aged ≤50 years (adjusted β = -0.025 per 0.001 µg/m³ Pb, 95% CI: -0.031 to -0.018, p < 0.001) compared with those >50 years (adjusted β = -0.015 per 0.001 µg/m³ Pb, 95% CI: -0.020 to -0.011, p < 0.001). Similarly, in adjusted models, females exhibited a larger decline in T-score (adjusted β = -0.022 per 0.001 µg/m³ Pb) than males (adjusted β = -0.012 per 0.001 µg/m³ Pb) (**Supplementary Table S3**).

## Discussion

This large-scale study of 32,239 participants over four years provides robust evidence that chronic environmental Pb exposure is independently associated with reduced BMD. Each 0.001 μg/m³ increase in airborne lead concentration was associated with a -0.013 decrease in baseline T-score and a -0.006 decline in T-score over time, based on validated Geo-AI exposure modeling and stepwise multivariable regression. The large sample size, comprehensive covariate adjustment, and high-resolution exposure data strengthen the reliability of these findings and highlight the importance of incorporating environmental risk factors into osteoporosis prevention strategies.

These findings align with and extend prior literature on osteoporosis and BMD decline. Schott et al. (1998) reported that women with femoral neck T-scores < -2.5 had a threefold higher risk of hip fractures, with an unadjusted incidence of 16.4 per 1,000 woman-years compared to 1.1 per 1,000 woman-years in those with T-scores ≥ -1, highlighting the strong clinical relevance of BMD as a fracture risk predictor 17. Similarly, the National Health and Nutrition Examination Survey (NHANES) III data showed that individuals in the highest quartile of blood lead levels had significantly lower femoral neck and lumbar spine BMD than those in the lowest quartile, with a particularly pronounced effect among postmenopausal women 18. Korrick et al. (2002) found that bone lead concentrations were inversely associated with BMD in the tibia and femur, and Campbell and Auinger (2007) reported a 1.97-fold increased risk of osteoporosis in adults with high blood lead levels, even after adjusting for age, race, BMI, and smoking status 5, 19. Compared to these studies, our investigation is distinguished by its significantly larger sample size (n = 32,239), its incorporation of both cross-sectional and longitudinal analyses, and the use of high-resolution Geo-AI modeling to estimate ambient Pb exposure rather than relying on blood or bone Pb concentrations alone. Importantly, we not only confirmed the inverse association between Pb exposure and BMD but also quantified the magnitude of T-score decline attributable to incremental increases in environmental Pb concentration.

Not only in Western countries, but also in Eastern nations where rapid industrialization and urban pollution pose substantial public health challenges, emerging evidence supports the association between airborne Pb exposure and reduced BMD. A study from China reported that elevated blood lead levels were significantly associated with lower femoral neck and lumbar spine BMD in both men and women, especially among individuals residing near industrial zones with high ambient Pb concentrations 20. In South Korea, a population-based analysis found that environmental lead exposure correlated with decreased calcaneal BMD and an increased risk of osteoporosis in postmenopausal women, with a clear dose response relationship 21. These findings from high exposure Asian populations align with our observations in Taiwan, reinforcing the broader relevance of environmental lead exposure as a modifiable risk factor for skeletal demineralization in polluted regions.

Stratified analyses revealed that the negative impact of lead on bone health was more pronounced in participants aged ≤50 years and in females. The stronger association in younger individuals may be attributed to inherently higher bone turnover rates, which make the skeleton more vulnerable to toxicant-induced remodeling disturbances 22. In females, especially during periods such as menopause, when bone resorption accelerates, lead may be mobilized from skeletal stores more readily, thus exacerbating its effects on bone loss 18, 23. These findings underscore potential age- and sex-specific susceptibility to lead-related bone demineralization and suggest the need for targeted prevention in these high-risk groups.

While numerous studies have demonstrated a negative association between Pb exposure and BMD, some investigations have reported conflicting or null findings. Khalil et al., using data from the NHANES 1999-2002 survey, found no statistically significant association between blood lead levels and BMD at the femoral neck or lumbar spine in postmenopausal women after adjusting for age, BMI, and other covariates 24. Similarly, a study conducted by Jain and Hu on older men found that while lead accumulated in bone tissue, it did not independently predict changes in BMD over time 25. These discrepancies may stem from differences in study design, exposure assessment (e.g., blood vs. bone lead levels), sample size, or population characteristics such as age, sex, nutritional status, and co-exposure to other environmental pollutants. In addition, the cross-sectional nature of many studies may limit their ability to detect long-term skeletal effects of chronic lead exposure. These inconsistencies underscore the need for longitudinal studies with precise exposure modeling and comprehensive adjustment for confounders to clarify the true relationship between environmental lead and osteoporosis risk.

This study draws from the western region of Taiwan, an area historically affected by industrial emissions from petrochemical and steel manufacturing, as well as high motorcycle density (over two million in 2021). While the Taiwanese government has made strides in air quality improvement through industrial upgrades, promotion of electric motorcycles, expansion of renewable energy, and reforestation, environmental Pb exposure remains a concern. Given these widespread environmental risks, we advocate for the incorporation of environmental exposure assessments, particularly Pb, into osteoporosis risk evaluation frameworks, especially for older adults.

The mechanisms by which Pb affects bone are biologically plausible and multifaceted, involving disruptions in calcium metabolism, bone cell function, matrix composition, and hormonal regulation. Pb competes with calcium ions, interferes with ion transport channels, and alters intracellular calcium handling, thereby disrupting calcium homeostasis 1, 8. It also impairs the function of bone cells: in osteoblasts, Pb inhibits osteocalcin synthesis and collagen production, while in osteoclasts, it enhances resorptive activity and alters hormonal responsiveness 2, 9. Moreover, Pb can substitute for calcium in the bone matrix, especially in calcium-binding proteins like calmodulin, which impairs hydroxyapatite formation and compromises bone mineralization and strength 7. Additionally, Pb interferes with the signaling of key bone-regulating hormones such as parathyroid hormone, vitamin D, and calcitonin, further disrupting normal bone remodeling processes 26.

Several methodological limitations should be acknowledged. First, the use of calcaneal QUS instead of dual-energy X-ray absorptiometry (DXA) may have affected measurement precision. Although QUS is less precise than DXA, it was selected for its cost-effectiveness, portability, and feasibility for large-scale population screening; importantly, its validity has been supported by previous studies 27, 28. Second, while our Geo-AI model provided high-resolution estimates of Pb exposure, and has been validated in multiple peer-reviewed studies, including research on particulate matter and osteoporosis 29, 30, some exposure misclassification remains possible. Third, the study did not include certain potentially influential confounders, such as dietary calcium intake, serum vitamin D levels, genetic predisposition, or cumulative lifetime Pb exposure. Lastly, the follow-up period (2018-2020) reflects a mid-term duration; extending this timeframe would provide greater insight into the long-term effects of Pb on skeletal health.

## Conclusion

This study reinforces the detrimental impact of chronic environmental Pb exposure on bone health, demonstrating significant associations with both lower baseline BMD and accelerated T-score decline over time. Public health policies should intensify efforts to mitigate Pb contamination and incorporate environmental risk profiling into osteoporosis prevention strategies.

## Supplementary Material

Supplementary figures and tables.

## Figures and Tables

**Figure 2:**
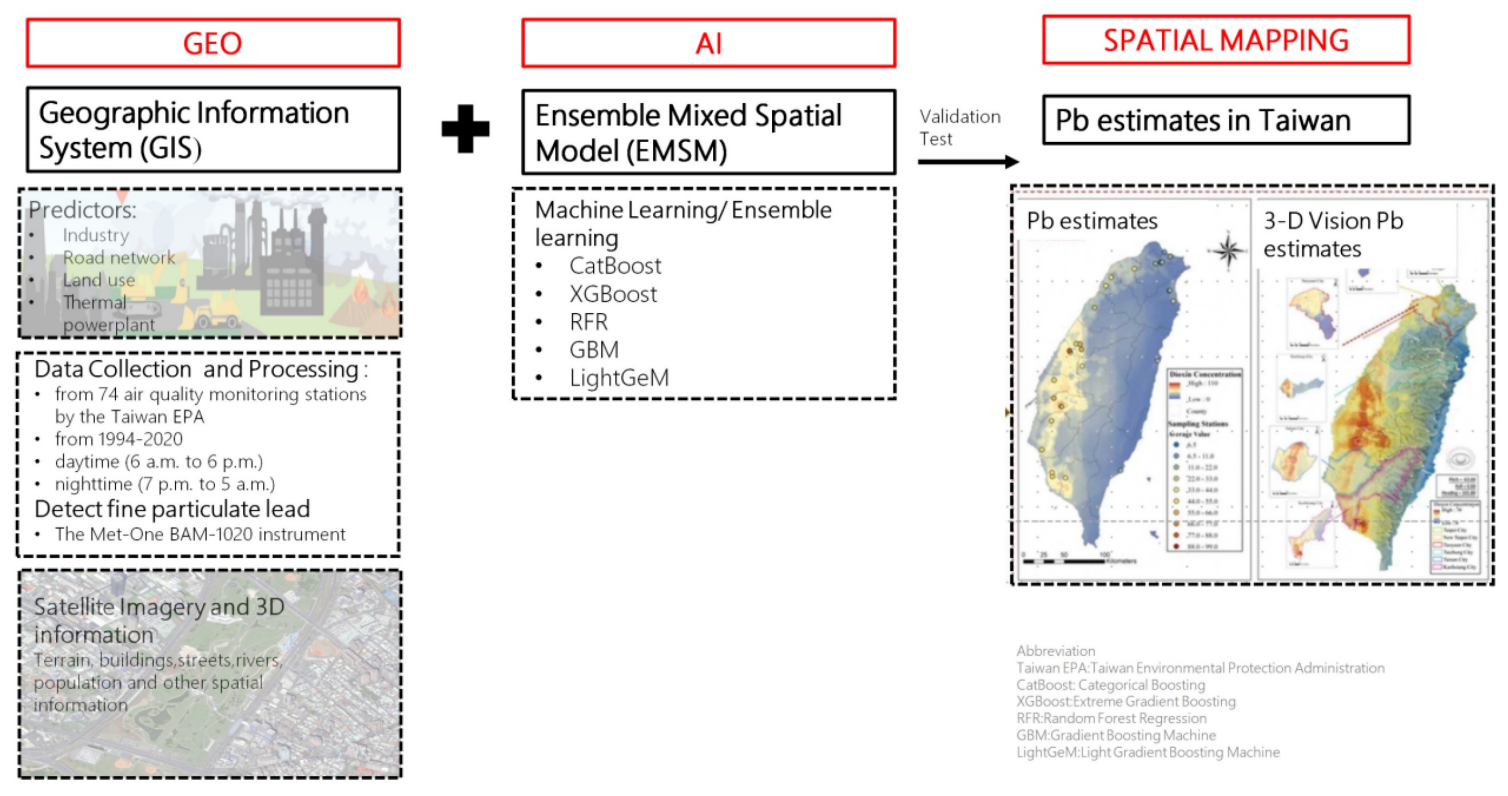
Workflow of the Geo-AI system for Pb estimation in Taiwan. This figure presents the Geo-AI system workflow, integrating Geographic Information System (GIS) data (e.g., industry, road networks, land use, thermal power plants) with ensemble machine learning models (CatBoost, XGBoost, RFR, GBM, LightGeM). Data from 74 Taiwan EPA air quality stations (1994-2020) and satellite imagery were used to map spatial and temporal lead (Pb) variations in both 2D and 3D visualizations.

**Table 1:** Demographic and clinical characteristics of study participants (n=32,239) stratified by bone density status (T-score)

Characteristic	All Participants (n=32,239)	T-score ≧ -2.5 (n=29,924)	T-score < -2.5 (n=2,315)	p
Pb exposure, ug/m3 (mean±SD)	0.00851±0.00400	0.00850±0.00399	0.00861±0.00407	0.194
T-score (mean±SD)	-0.3±1.6	-0.1±1.5	-3.1±0.5	<.0001
**Demographic data**				
Age (mean±SD)	49.7±11.3	49.1±11.2	58.1±9.2	<.0001
Female, n (%)	20,654 (64.1)	19,262 (64.4)	1,392 (60.1)	<.0001
Education, university and above, n (%)	21,458 (66.6)	20,291 (67.8)	1,167 (50.4)	<.0001
Regular Exercise, n (%)	12,419 (38.5)	11,321 (37.8)	1,098 (47.4)	<.0001
WC, cm (mean±SD)	83.4±10.4	83.4±10.4	83.4±10.7	0.679
BMI, kg/m2 (mean±SD)	24.3±3.9	24.3±3.9	23.6±3.8	<.0001
Smoking, current, n (%)	8,896 (27.6)	8,207 (27.4)	689 (29.8)	0.016
Alcohol, current, n (%)	2,070 (6.4)	1,928 (6.4)	142 (6.1)	0.597
**Comorbidities**				
Diabetes, n (%)	1,686 (5.2)	1,517 (5.1)	169 (7.3)	<.0001
Hypertension, n (%)	3,783 (11.7)	3,377 (11.3)	406 (17.5)	<.0001
Dyslipidemia, n (%)	2,553 (7.9)	2,310 (7.7)	243 (10.5)	<.0001
Cataract, n (%)	3,240 (10.1)	2,757 (9.2)	483 (20.9)	<.0001
**Laboratory data**				
SBP (mean±SD)	122.5±19.0	122.1±19.0	127.7±19.2	<.0001
DBP (mean±SD)	74.6±11.6	74.5±11.6	75.7±11.7	<.0001
TC (mean±SD)	196.4±36.5	196.1±36.3	200.8±38.1	<.0001
Albumin (mean±SD)	4.5±0.2	4.50.2	4.5±0.2	<.0001
Hb (mean±SD)	13.8±1.6	13.8±1.6	13.91.4	0.070
Uric acid (mean±SD)	5.3±1.4	5.3±1.4	5.4±1.4	0.357

Abbreviations: Pb, blood lead level; WC, waist circumference; BMI, body Mass Index; SBP, systolic blood pressure; DBP, diastolic blood pressure; TC, total cholesterol; Hb, hemoglobin; SD, standard Deviation.

**Table 2:** Parameters associated with T-score in univariable linear regression analysis.

Parameters	β	95%CI		p
Pb (per 0.001 ug/m^3^)	-0.008	-0.013	-0.004	0.0003
Demographic data				
Age (per 1 year)	-0.057	-0.058	-0.055	<0.0001
Female (vs. male)	0.433	0.396	0.469	<0.0001
Education, university and above (vs. no)	0.586	0.549	0.623	<0.0001
Regular Exercise (vs. no)	-0.227	-0.264	-0.190	<0.0001
WC (per 1 cm)	-0.005	-0.007	-0.003	<0.0001
BMI (per 1 kg/m^2^)	0.030	0.025	0.034	<0.0001
Smoking, current (vs. no)	-0.209	-0.249	-0.170	<0.0001
Alcohol, current (vs. no)	-0.139	-0.211	-0.066	0.0002
Comorbidities				
Diabetes (vs. no)	-0.398	-0.478	-0.318	<0.0001
Hypertension (vs. no)	-0.548	-0.603	-0.493	<0.0001
Dyslipidemia (vs. no)	-0.499	-0.563	-0.434	<0.0001
Cataract (vs. no)	-0.799	-0.858	-0.740	<0.0001
Laboratory data				
SBP (per 1 mmHg)	-0.014	-0.015	-0.013	<0.0001
DBP (per 1 mmHg)	-0.012	-0.014	-0.011	<0.0001
TC (per 1 mg/dl)	-0.003	-0.004	-0.003	<0.0001
Albumin (per 1 g/dl)	0.093	0.017	0.170	0.0171
Hb (per 1 g/dl)	-0.102	-0.113	-0.091	<0.0001
Uric acid (per 1 mg/dl)	-0.076	-0.089	-0.064	<0.0001

Abbreviations: Pb, blood lead level; WC, waist circumference; BMI, body Mass Index; SBP, systolic blood pressure; DBP, diastolic blood pressure; TC, total cholesterol; Hb, hemoglobin; CI, Confidence interval.

**Table 3:** Parameters associated with T-score in multivariable linear regression analysis.

Multivariable regression (Sig. from Univariable)					Stepwise			
Parameters	β	95%CI		p	β	95%CI		p
Pb (per 0.001 ug/m^3^)	-0.013	-0.017	-0.009	<0.0001	-0.013	-0.017	-0.009	<0.0001
Demographic data								
Age (per 1 year)	-0.054	-0.056	-0.052	<0.0001	-0.054	-0.056	-0.052	<.0001
Female (vs. male)	0.488	0.437	0.539	<0.0001	0.468	0.430	0.507	<.0001
Education, university and above	0.261	0.225	0.297	<0.0001	0.258	0.222	0.294	<.0001
Regular exercise (vs. no)	0.251	0.216	0.286	<0.0001	0.250	0.216	0.285	<.0001
WC (per 1 cm)	-0.023	-0.027	-0.020	<0.0001	-0.023	-0.027	-0.020	<.0001
BMI (per 1 kg/m^2^)	0.102	0.094	0.111	<0.0001	0.102	0.093	0.110	<.0001
Smoking, current (vs. no)	0.009	-0.032	0.051	0.6612	-	-	-	-
Alcohol, current (vs. no)	0.055	-0.014	0.123	0.1177	-	-	-	-
Comorbidities								
Diabetes (vs. no)	0.108	0.031	0.185	0.0058	0.106	0.031	0.181	0.0055
Hypertension (vs. no)	-0.020	-0.077	0.036	0.4873	-	-	-	-
Dyslipidemia (vs. no)	0.004	-0.060	0.068	0.9062	-	-	-	-
Cataract (vs. no)	-0.082	-0.139	-0.025	0.0048	-0.084	-0.141	-0.027	0.0037
Laboratory data								
SBP (per 1 mmHg)	-0.002	-0.003	-0.000	0.0243	-0.002	-0.003	-0.001	0.0031
DBP (per 1 mmHg)	0.001	-0.002	0.003	0.6487	-	-	-	-
TC (per 1 mg/dl)	-0.001	-0.002	-0.001	<0.0001	-0.001	-0.001	-0.001	<0.0001
Albumin (per 1 g/dl)	-0.122	-0.198	-0.047	0.0015	-0.115	-0.188	-0.042	0.0021
Hemoglobin (per 1 g/dl)	0.010	-0.004	0.023	0.1512	-	-	-	-
Uric acid (per 1 mg/dl)	-0.008	-0.022	0.007	0.3080	-	-	-	-

Abbreviations: Pb, blood lead level; WC, waist circumference; BMI, body Mass Index; SBP, systolic blood pressure; DBP, diastolic blood pressure; TC, total cholesterol; Hb, hemoglobin; CI, Confidence interval.

**Table 4:** Linear and stepwise multivariable regression results for T-score change with lead exposure, demographics, comorbidities, and laboratory data. Estimates (β), T-scores, 95% CI, and P-values are presented.

Univariable regression					Stepwise multivariable regression			
Parameters	β (ΔT-score)	95%CI		p	β (ΔT-score)	95%CI		p
Pb (per 0.001 ug/m3)	-0.003	-0.006	0.000	0.0847	-0.006	-0.010	-0.003	0.0002
Follow-up time (per 1 year)	-0.031	-0.045	-0.016	<0.0001	-0.050	-0.066	-0.035	<0.0001
Demographic data								
Age (per 1 year)	-0.006	-0.008	-0.005	<0.0001	-0.005	-0.007	-0.004	<0.0001
Female (vs. male)	-0.101	-0.129	-0.073	<0.0001	-0.098	-0.128	-0.068	<0.0001
Education, university and above	0.084	0.057	0.110	<0.0001	0.037	0.008	0.066	0.0122
Regular exercise (vs. no)	-0.077	-0.103	-0.050	<0.0001	-0.033	-0.062	-0.004	0.0254
WC (per 1 cm)	0.003	0.001	0.004	<0.0001	-	-	-	-
BMI (per 1 kg/m2)	0.010	0.006	0.014	<0.0001	0.009	0.005	0.013	<0.0001
Smoking, current (vs. no)	0.079	0.048	0.110	<0.0001	-	-	-	-
Alcohol, current (vs. no)	0.060	0.002	0.118	0.0425	-	-	-	-
Comorbidities								
Diabetes (vs. no)	0.041	-0.019	0.100	0.1780	-	-	-	-
Hypertension (vs. no)	0.011	-0.028	0.050	0.5728	0.045	0.002	0.088	0.0400
Dyslipidemia (vs. no)	-0.024	-0.074	0.025	0.3364	-	-	-	-
Cataract (vs. no)	-0.041	-0.088	0.005	0.0830	-	-	-	-
Laboratory data								
SBP (per 1 mmHg)	-0.002	-0.002	-0.001	0.0001	-0.001	-0.002	-0.000	0.0088
DBP (per 1 mmHg)	-0.0002	-0.001	0.001	0.7302	-	-	-	-
TC (per 1 mg/dl)	-0.001	-0.002	-0.001	<0.0001	-0.001	-0.001	-0.000	<0.0001
Albumin (per 1 g/dl)	-0.076	-0.136	-0.016	0.0127	-0.141	-0.204	-0.078	<0.0001
Hb (per 1 g/dl)	0.010	0.001	0.018	0.0301	-	-	-	-
Uric acid (per 1 mg/dl)	0.021	0.011	0.030	<0.0001	-	-	-	-

Abbreviations: Pb, blood lead level; WC, waist circumference; BMI, body Mass Index; SBP, systolic blood pressure; DBP, diastolic blood pressure; TC, total cholesterol; Hb, hemoglobin; CI, Confidence interval.
