# Supplementary material for: Environmental Lead Exposure Predicts Lower Bone Mineral Density in a Large Taiwanese Population-Based Study
Source: Int J Med Sci. 2025 Sep 8;22(15):3985–93. doi: 10.7150/ijms.117042 (PMC12492357; doi:10.7150/ijms.117042)
Supplement: Supplementary file 1 — Supplementary figures and tables. [file ijmsv22p3985s1.pdf]

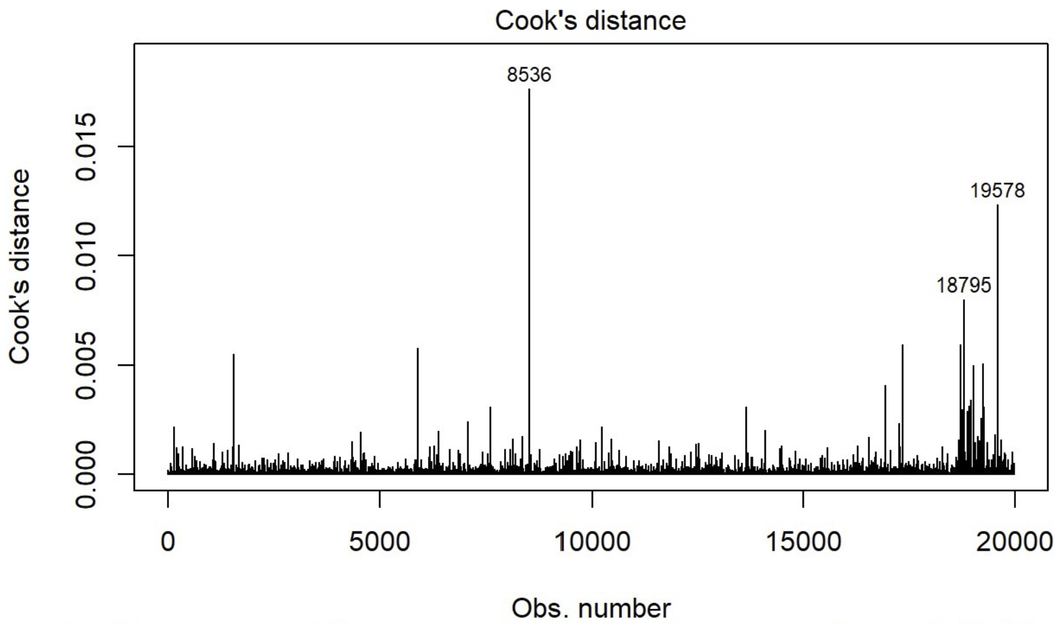

Supplementary Figure S1. Cook's distance plot for influential observations. Each point represents an observation; all Cook's distance values were < 0.02, indicating no single observation had undue influence on the regression model.

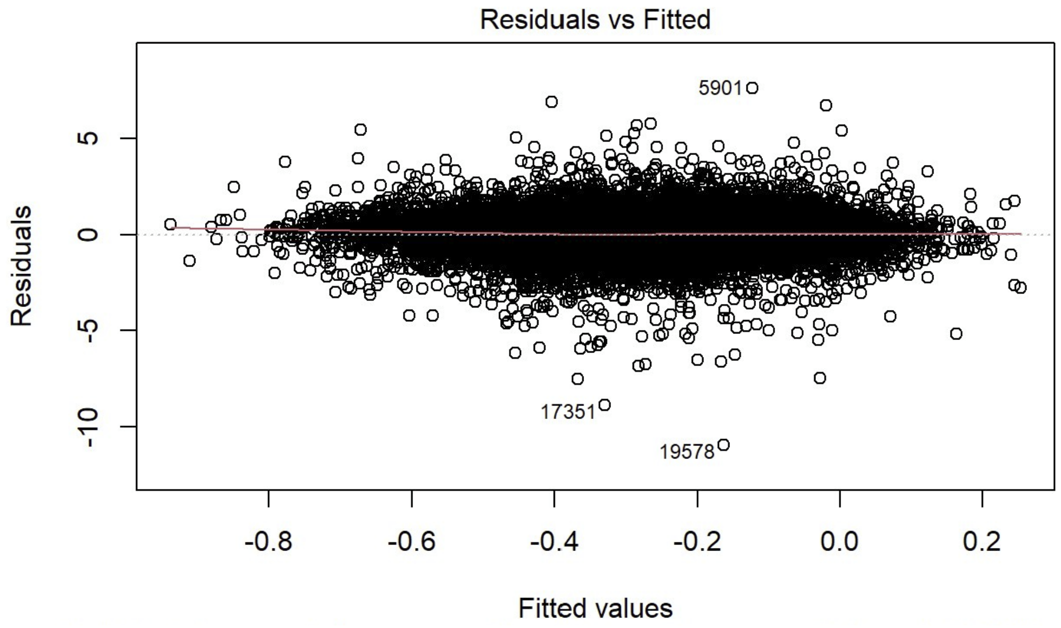

Supplementary Figure S2. Residuals vs. fitted values plot. Displays standardized residuals against fitted values to assess linearity and homoscedasticity. The absence of clear patterns supports that model assumptions are reasonably met.

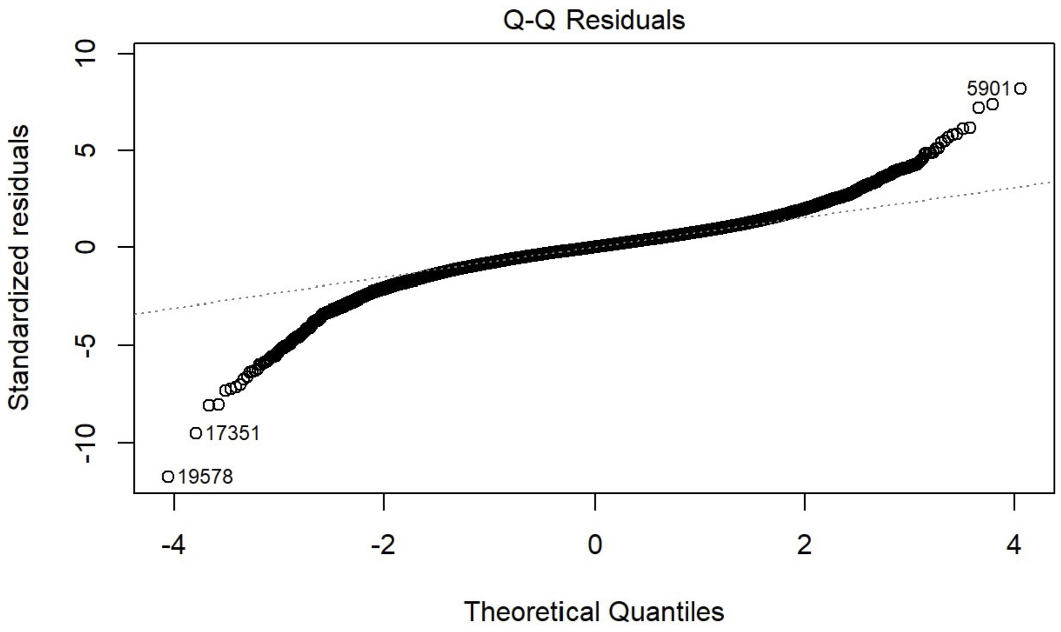

Supplementary Figure S3. Q-Q plot of standardized residuals. Compares the distribution of standardized residuals to the theoretical normal distribution. Slight deviations in the tails were observed, but overall normality was acceptable for large-sample inference.

Variable	β	Std. Error	t value	p
(Intercept)	1.610	0.176	9.173	<0.001
Pb	-19.018	1.933	-9.840	<0.001
Follow-up time	-0.014	0.001	-15.602	<0.001
Age	-0.008	0.001	-11.753	<0.001
Sex (female = 1)	0.125	0.018	7.092	<0.001
Education	-0.036	0.026	-1.421	0.155
Body mass index	0.005	0.002	2.327	0.020
Diabetes (yes = 1)	0.068	0.032	2.151	0.032
Cataract (yes = 1)	0.036	0.025	1.454	0.146
Albumin	-0.167	0.032	-5.159	<0.001
Hemoglobin	-0.010	0.006	-1.660	0.097
Breusch-Pagan (BP) test	140.260	-	-	< 0.001

Supplementary Table S1. Stepwise multivariable regression with heteroscedasticity-consistent (HC3) robust standard errors. Regression coefficients (β), standard errors, t values, and p-values are shown. HC3 heteroscedasticity-consistent standard errors were used due to heteroscedasticity detected by the Breusch-Pagan (BP) test (BP statistic = 140.260, $p < 0.001$).

Variable	GVIF	GVIF^(1/(2*Df))
Pb	1.123	1.060
Follow time	1.118	1.057
Age	1.315	1.147
Sex	1.680	1.296
Education	1.213	1.050
Body mass index	1.101	1.049
Diabetes	1.058	1.028
Cataract	1.114	1.056
Albumin	1.142	1.069
Hemoglobin	1.737	1.318

Supplementary Table S2. Variance inflation factor (VIF) analysis for variables retained in the final stepwise multivariable regression model of T-score change. This table lists each predictor included in the final stepwise multivariable regression model, along with its variance inflation factor (VIF) or generalized VIF (GVIF) for categorical variables. GVIF values are adjusted using GVIF^(1/(2×Df)) to allow comparison with standard VIF values. Df indicates the degrees of freedom for each variable (numeric variables = 1; categorical variables = number of categories - 1). All VIF values were < 2, confirming that multicollinearity was not a concern.

Stratum	Model	β (ΔT-score per 0.001 μg/m³ Pb)	95% CI Lower	95% CI Upper	p
Age group					
>50 years	Unadjusted	-0.006	-0.010	-0.001	0.014
≤50 years	Unadjusted	-0.014	-0.020	-0.008	<0.001
>50 years	Adjusted	-0.015	-0.020	-0.011	<0.001
≤50 years	Adjusted	-0.025	-0.031	-0.018	<0.001
Sex					
Female	Unadjusted	-0.012	-0.017	-0.007	<0.001
Male	Unadjusted	-0.004	-0.010	0.001	0.130
Female	Adjusted	-0.022	-0.027	-0.017	<0.001
Male	Adjusted	-0.012	-0.018	-0.006	<0.001

Supplementary Table S3. Stratified analysis of the association between lead exposure and T-score change, by age group and sex. β represents the regression coefficient for the change in T-score (ΔT-score) per 0.001 μg/m³ increase in air lead exposure. Adjusted models include age, sex, follow-up time, body mass index, albumin, and comorbidities as covariates.
